# Nicotinamide mononucleotide (NMN) alleviates the poly(I:C)-induced inflammatory response in human primary cell cultures

**DOI:** 10.1038/s41598-023-38762-x

**Published:** 2023-07-20

**Authors:** Hitomi Sano, Anton Kratz, Taiko Nishino, Haruna Imamura, Yuki Yoshida, Noriaki Shimizu, Hiroaki Kitano, Ayako Yachie

**Affiliations:** 1 The Systems Biology Institute, Saisei Ikedayama Bldg., 5-10-25, Higashi Gotanda, Shinagawa-ku, Tokyo, 141-0022 Japan; 2 Ginza Research Center, Mirailab Bioscience Inc., 6F Prairie Ginza Bldg., 1-14-4, Ginza, Chuo-ku, Tokyo, 104-0061 Japan; 3 SBX BioSciences, Inc., 1600 - 925 West Georgia Street, Vancouver, BC V6C 3L2 Canada

**Keywords:** Bayesian inference, Regulatory networks, Computational biology and bioinformatics

## Abstract

NMN is the direct precursor of nicotinamide adenine dinucleotide (NAD+) and is considered as a key factor for increasing NAD+ levels and mitochondrial activity in cells. In this study, based on transcriptome analysis, we showed that NMN alleviates the poly(I:C)-induced inflammatory response in cultures of two types of human primary cells, human pulmonary microvascular endothelial cells (HPMECs) and human coronary artery endothelial cells (HCAECs). Major inflammatory mediators, including *IL6* and PARP family members, were grouped into coexpressed gene modules and significantly downregulated under NMN exposure in poly(I:C)-activated conditions in both cell types. The Bayesian network analysis of module hub genes predicted common genes, including eukaryotic translation initiation factor 4B (*EIF4B*), and distinct genes, such as platelet-derived growth factor binding molecules, in HCAECs, which potentially regulate the identified inflammation modules. These results suggest a robust regulatory mechanism by which NMN alleviates inflammatory pathway activation, which may open up the possibility of a new role for NMN replenishment in the treatment of chronic or acute inflammation.

## Introduction

Nicotinamide mononucleotide (NMN) is the direct precursor of nicotinamide adenine dinucleotide (NAD+) and is considered a key factor for increasing NAD+ levels in cells; NAD+ plays an especially active role in metabolic processes, such as glycolysis, the TCA cycle, and the electron transport chain. A study in aged mice and *Caenorhabditis elegans* showed an age-associated reduction in NAD+ levels and demonstrated that the protein deacetylase *sir-2.1*-dependent activation of the mitochondrial unfolded protein response and the activation of the FOXO transcription factor DAF-16 are crucial for longevity in worms^1^. NMN has been shown to mitigate age-associated physical decline in wild-type mice as evidenced by many phenotypic effects, including reduction of body weight, enhanced energy metabolism, as well as improved insulin sensitivity and plasma lipid profiles^2^. Several NAD+ precursors, nicotinamide riboside (NR), NMN, and dihydronicotinamide riboside (NRH), were tested in both preclinical and clinical studies^3^. NMN/NR were shown to induce mitophagy, and impaired mitophagy was considered to accelerate aging in Werner syndrome and neurodegeneration in Alzheimer’s disease^4,5^. Enhancement of metabolic processes via elevation of cellular NAD+ levels is an evolutionarily conserved cellular stress response across various species. In addition, preclinical and clinical trials of the administration of several NAD+ precursors to humans are in progress. Recently, an in vivo mouse study showed that NAD+ supplementation may protect the lung from inflammatory injury, including cell death, caused by SARS-CoV-2 infection in both aged and young mice^6^. The idea that PARPs consume NAD+ to catalyze ADP-ribosylation (ADPR) in the context of antiviral processes while viral proteins attempt to undo ADPR, thereby increasing the demand for NAD+, has been conceptualized as a “NAD+ battlefield”^7^. Thus, the detailed molecular mechanism by which NAD+ supplementation alleviates the inflammatory response in various human cell types must be elucidated.

In this paper, we studied the effects of NMN on gene expression profiles in cultures of two types of human primary cells, human pulmonary microvascular endothelial cells (HPMECs) and human coronary artery endothelial cells (HCAECs), under exposure to polyinosinic-polycytidylic acid (poly(I:C)). Poly(I:C), a synthetic analog of double-stranded RNA (dsRNA) present in some viruses, is used to model the effects of extracellular dsRNA to mimic the inflammatory response induced by viral infection^8^. Poly(I:C) is recognized by endosomal Toll-like receptor 3 (*TLR3*)^9,10^. Upon recognition of poly(I:C), *TLR3* activates the transcription factor interferon regulatory factor 3 (*IRF3*)^11^, which then leads to the production of type I IFNs (*IFNB1*). A systems biology study with human data from cohorts of patients with a diagnosis of SARS-CoV-2, influenza, or respiratory syncytial virus (RSV) indicated that the expression levels of 43 cytokines, including *IL6*, were significantly upregulated^12^. A recent study showed that NMN supplementation suppressed proinflammatory cytokine production by repairing altered NAD+ metabolism in activated macrophages under exposure to the bacterial endotoxin lipopolysaccharide (LPS), which is also often used as a model to study the inflammatory response^13^.

In this study, we show that NMN alleviates poly(I:C)-induced increases in the expression of inflammatory response-related factors, including *IL6* and PARP family members. Genes with similar expression profiles were grouped via weighted gene correlation network analysis (WGCNA)^14^, and important functional modules related to the inflammatory response as well as mitochondrial metabolism were identified. Furthermore, causal network inferences of the responses using Bayesian network analysis showed that modules enriched in key inflammation genes, including *IL6* and PARP family members, which were upregulated by poly(I:C) and downregulated by NMN, were predicted to be regulated by gene modules enriched in translational regulation-related genes, including eukaryotic translation initiation factor 4B (*EIF4B*) and ribosomal proteins common to both cell lines. Furthermore, the modules enriched in platelet-derived growth factor binding genes and blood vessel development-related genes were identified as potential upstream regulators of the poly(I:C)-induced inflammatory response.

## Methods

### Human primary cell cultures

Human pulmonary microvascular endothelial cells (HPMECs) and human coronary artery endothelial cells (HCAECs) were obtained from ScienCell Research Laboratories and maintained in endothelial cell medium (ScienCell Research Laboratories, Carlsbad, USA) in a humidified atmosphere at 37 °C with 5% CO_2_. The culture dishes and plates were precoated with fibronectin at a concentration of 2 μg/cm^2^. After the frozen vial was thawed in a 37° water bath, the entire sample was seeded in one 100 mm dish, followed by changing the medium every 2–3 days, and the cells were cultured until sufficient cell numbers were obtained. Cells were detached with 0.05% trypsin EDTA and subcultured, and expansion culture by subculturing was continued until the number of cells required for the test was obtained. Both cell lines were passaged twice after thawing, and the cells were spread onto 12-well tissue culture-treated plates and cultured for 2 days. For RT‒PCR, cells were treated with polyinosinic-polycytidylic acid (poly(I:C); Enzo Life Sciences, Inc.) at final concentrations of 0, 2.5, and 25 µg/ml for 6 h or 9 h. Total RNA was prepared with an RNeasy Plus Mini Kit (QIAGEN) and subjected to reverse transcription with a SuperScript VILO cDNA Synthesis Kit (Thermo Fisher Scientific) to obtain cDNA. Quantitative PCR was performed with TaqMan Gene Expression Master Mix, PowerUp SYBR Green Master Mix, and TaqMan Gene Expression Assays (Thermo Fisher Scientific). The list of primers used is shown in Supplementary Table 1. For transcriptome analysis, cells were treated with or without 25 µg/ml poly(I:C) in a CO_2_ incubator for 6 h and further cultured in fresh medium containing 0, 0.1 (low), 1.0 (medium), or 10.0 (high) mM NMN for 48 h. NMN was provided by Mirailab Bioscience Inc. (Tokyo, Japan). In the group subjected to poly(I:C) induction, the supernatants from the 6-well plates seeded with cells were removed and replaced with poly(I:C)-containing medium. On the other hand, in the group that was not subjected to poly(I:C) induction, poly(I:C) was replaced with medium containing water. After induction with poly(I:C) in a CO_2_ incubator for 6 h, the supernatant was removed, the medium was replaced with fresh medium containing 0, 0.1 (low), 1.0 (medium), or 10.0 (high) mM NMN, and the cells were further cultured for 48 h in a CO_2_ incubator. Cells from 3 independent cultures under 8 different conditions (4 doses of NMN with or without poly(I:C) induction, as described above) were subjected to total RNA preparation using TRI Reagent (Molecular Research Center, Inc.) to generate triplicate samples. The cells were approximately 40–50% confluent at the time of poly(I:C) induction and 100% confluent 48 h after the addition of NMN. Images of representative cells from each condition at the time of RNA isolation are shown in Supplementary Fig. 1.

### RNA-seq and RNA-seq data analysis

Library construction for whole transcriptome sequencing was performed using the TruSeq Stranded mRNA LT Sample Prep Kit (Illumina, San Diego, CA) according to the manufacturer's protocols^15^. Transcriptome library sequencing was carried out using the 101-bp paired-end mode with Illumina NovaSeq 6000. The data integrity of the raw sequences was evaluated with FastQC v0.11.7 (http://www.bioinformatics.babraham.ac.uk/projects/fastqc/), and the sequences were trimmed with Trimmomatic 0.38 (http://www.usadellab.org/cms/?page=trimmomatic) to remove reads with lengths shorter than 36 bp. The trimmed reads were mapped against the reference genome GRCh38 using HISAT2 version 2.1.0 (https://ccb.jhu.edu/software/hisat2/index.shtml) and Bowtie2 2.3.4.1 aligner. The abundance of genes/transcripts was calculated from the read count and normalized as FPKM (Fragments Per Kilobase of transcript per Million mapped reads) for each sample (Data Availability). Sample preparation and RNA-seq analysis were performed by Cosmobio Co. (Sapporo, Japan).

### Principal component analysis

For principal component analysis (PCA) of transcriptome data, the PCA function implemented as part of *sklearn.decomposition* in Python was utilized^16^. The potential outliers were removed from further analysis.

### Differentially expressed gene analysis

Differentially expressed genes (DEGs) between the control and high-dose (10 mM) NMN groups were identified using R version 4.1.0 (2021–05-18); the *fdrtool* library^17^ was utilized to compute the adjusted p value based on the false discovery rate (FDR). The thresholds for log2-fold change (FC), p value, and local FDR were set to 1.0 (twofold), 0.05, and 0.1, respectively.

### Weighted gene correlation network analysis

The *WGCNA* package (v 1.70) in R (4.1.0, 2021–05–18) was used to cluster all genes from the transcriptome data according to their expression levels among different doses of NMN under non-poly(I:C)-activated or poly(I:C)-activated conditions. The minimum module size was set to 30, and the soft power for scale-free topology model fit was set to 0.9. Eigengenes for identified modules were identified with the *moduleEigengenes()* function, which calculates the first principal component for each module. The correlations of the computed module eigengenes and gene expression were then calculated to quantify the similarity of all genes included in the modules.

### Pathway enrichment analysis

Pathway definitions were obtained from Gene Ontology (GO) (retrieved October 17, 2020). For each module identified with WGCNA, overrepresentation of genes in pathways was tested based on the pathway definitions using the Benjamini and Hochberg^18^ approach for FDR to calculate the adjusted p value.

### Bayesian network analysis

Causal network prediction was conducted by utilizing a general Bayesian network in which each node models the expression levels of the eigengenes^19^. The *bn.boot()* function of the *bnlearn* package (version 4.6.2) was used to fit directed acyclic graphs (DAGs) in Bayesian network structures to the expression levels of the eigengenes. To obtain a consensus network, network learning via *bn.boot* was conducted 500 times with three different hybrid structure learning algorithms, Max–Min Hill Climbing (MMHC), Hybrid HPC (H2PC), and the more general 2-phase Restricted Maximization (RSMAX2) algorithms. The strength and directionality of each edge in each algorithm were computed with *boot.strength()* function as the probability of observing edge in the bootstrap replicates (ranging from 0.0 to 1.0), regardless of its direction, and as the probability of that particular direction in the bootstrap replicates conditional on the presence of the edge (ranging from 0.0 to 1.0), respectively. The edges with strength > 0.1 as well as direction > 0.5 were taken as valid directed edges. The consensus network illustrated in Fig. 4 was constructed by taking the sum of the strength computed via the three algorithms, so that the strength should be ranging from 0.0 to 3.0.

## Results

**Figure 1:**
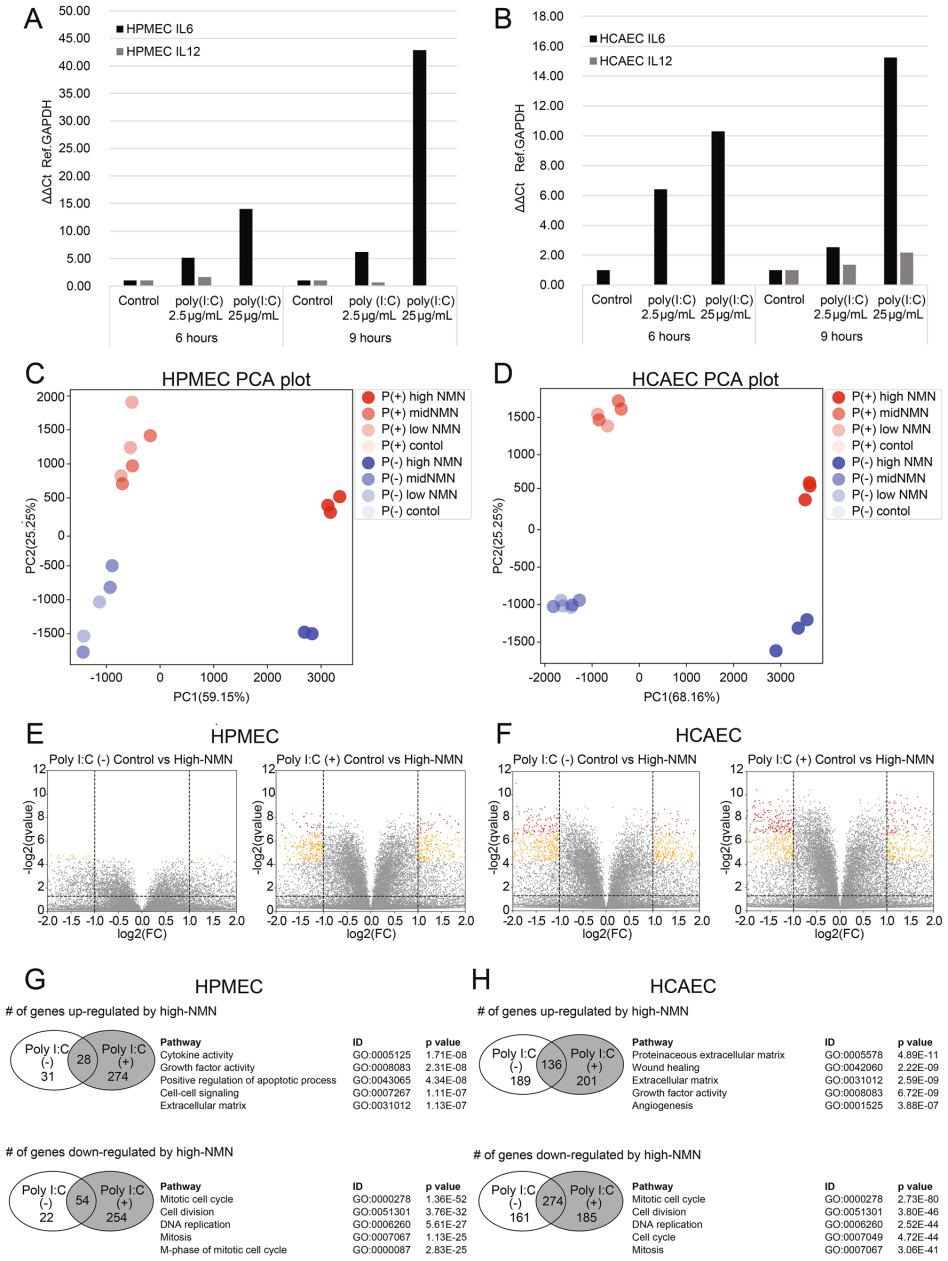
Changes in gene expression levels according to poly(I:C) activation condition and NMN dose. *IL6* and *IL12* expression levels after control, poly(I:C) (2.5 µg/mL), and poly(I:C) (25 µg/mL) treatment for 6 h and 9 h in HPMECs (**A**) and in HCAECs (**B**). PCA plot of expression profiles after poly(I:C) (2.5 µg/mL) activation (red) and without poly(I:C) activation (blue) in HPMECs (**C**) and HCAECs (**D**). Volcano plots of differentially expressed genes (DEGs) between control and high-dose NMN treatments in non-poly(I:C)-activated and poly(I:C)-activated HPMECs (**E**) and non-poly(I:C)-activated and poly(I:C)-activated HPMECs (**F**). Number of genes upregulated or downregulated under poly(I:C)-activated conditions via high-dose (10 mM) NMN and list of pathways overrepresented in HPMECs (**G**) and HCAECs (**H**).

First, we confirmed by RT‒PCR that the expression levels of *IL6* and *IL12* mRNA were significantly increased after poly(I:C) activation in both HPMECs (Fig. 1A) and HCAECs (Fig. 1B). *IL6* is the major inflammation-associated cytokine that is significantly and robustly upregulated during viral infections, such as SARS-CoV-2, influenza, and RSV infections^12^. Increases in both *IL6* and *IL12* mRNA expression levels indicate that the addition of poly(I:C) to the culture medium successfully induced an inflammatory response in the cells. We then tested the effect of NMN treatment on the inflammatory response induced by poly(I:C). The transcriptome data were obtained from three samples of each of the eight different conditions, non-poly(I:C)-activated or poly(I:C)-activated with three different NMN dose levels (0.1 mM, 1.0 mM, and 10.0 mM) and controls for HPMECs and HCAECs. Notably, samples treated with high NMN doses were aggregated into a small cluster regardless of poly(I:C) activation for both HPMECs and HCAECs, suggesting that high-dose NMN resulted in a similar gene expression profile compared to low-dose and medium-dose NMN.

Principal component analysis (PCA) was performed to provide a broad view of the transcriptome data for HPMECs and HCAECs. In HPMECs, one low-NMN (0.1 mM) replicate under the non-poly(I:C)-activated condition and one high-NMN (10.0 mM) replicate from the non-poly(I:C)-activated condition were apparent outliers; similarly, one low-NMN (0.1 mM) replicate from the poly(I:C)-activated condition was an exception in HCAECs (Supplementary Fig. 2). Those apparent outliers were removed from further analysis. Figure 1C,D shows the PCA results without these outliers. In both HPMECs and HCAECs, principal component 1 (PC1) distinguishes high-NMN samples from other samples, and PC2 distinguishes non-poly(I:C)-activated from poly(I:C)-activated conditions. The genes that contribute to PC1 and PC2 in the HPMEC and HCAEC transcriptomes are represented as gene loadings in Supplementary Table 2. In addition, high-NMN samples were densely gathered in the PCA plot both with and without poly(I:C) activation, suggesting distinct and unique expression changes in cell cultures with high concentrations of NMN.

To determine the distinct characteristics of samples treated with high-dose NMN, differentially expressed genes (DEGs) between control and high-NMN (10 mM) samples were identified for both non-poly(I:C)-activated and poly(I:C)-activated conditions in HPMECs (Fig. 1E) and HCAECs (Fig. 1F). In HPMECs, the number of DEGs between the control and high-NMN groups was small under non-poly(I:C)-activated conditions, with 59 upregulated and 76 downregulated genes, but the number was large under poly(I:C)-activated conditions, with 302 upregulated and 308 downregulated genes (Fig. 1G); this result indicates that high-dose application of NMN is more effective for poly(I:C)-activated conditions than for non-poly(I:C)-activated conditions in HPMECs. On the other hand, the number of DEGs was relatively large regardless of poly(I:C) activation in HCAECs (Fig. 1H). In both HPMECs and HCAECs, pathways related to extracellular matrix and cell population proliferation were overrepresented among genes upregulated by high NMN under poly(I:C)-activated conditions, and pathways related to mitotic cell cycle, cell division, and chromosome segregation/organization were overrepresented in genes downregulated by high NMN under poly(I:C)-activated conditions. Supplementary Information 1 lists the top 20 genes that were upregulated or downregulated by high NMN under poly(I:C)-activated conditions. Supplementary Information 2 contains the complete list of Gene Ontology pathways enriched in up- and downregulated genes of HPMEC and HCAEC.

**Figure 2:**
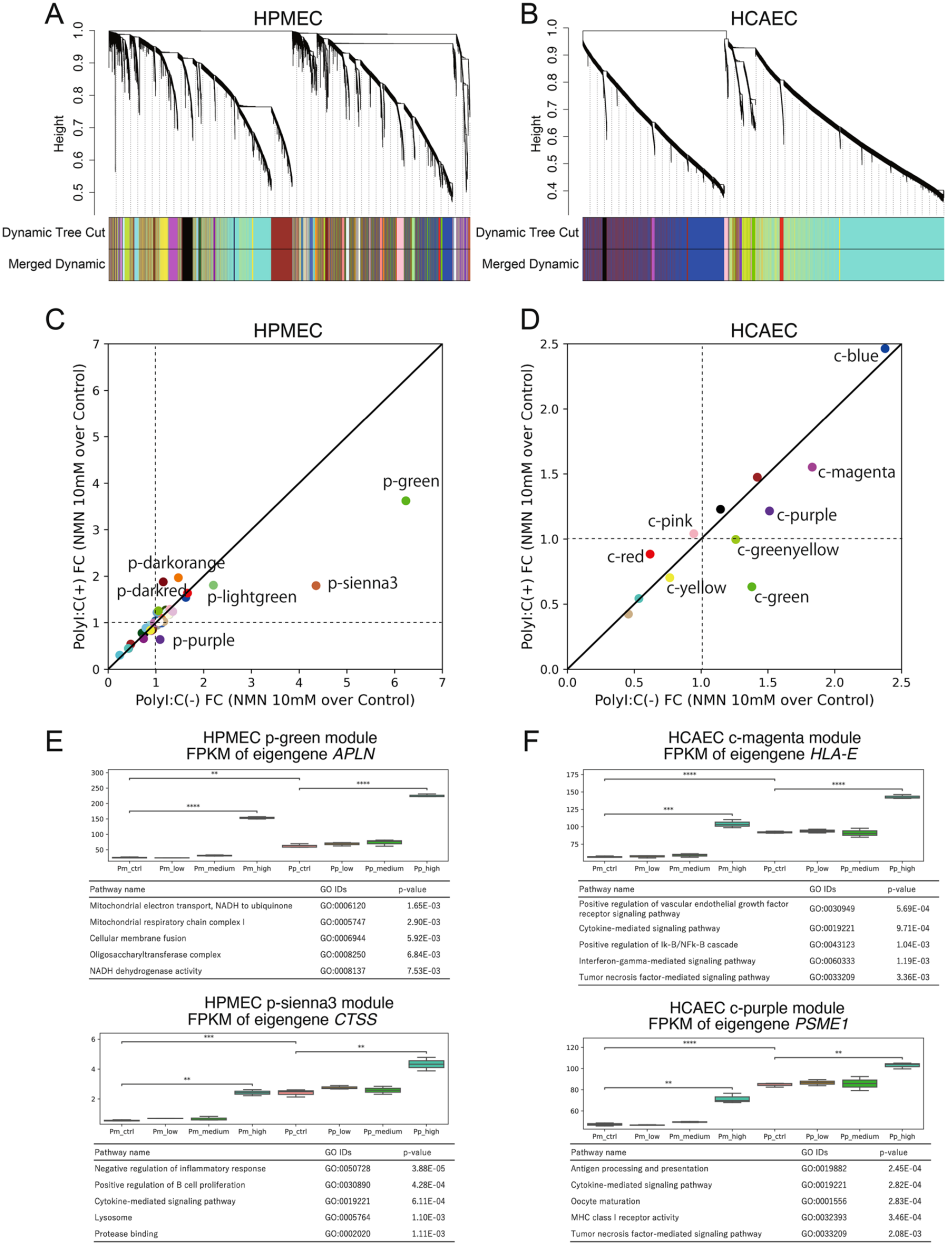
Clustering transcriptome data via weighted gene correlation network analysis (WGCNA). Dendrograms of all genes and modules with different colors for HPMECs (**A**) and HCAECs (**B**). Scatter plots of the fold change (FC) of the eigengenes of identified modules from control to high-dose (10 mM) NMN in the non-poly(I:C)-activated condition (x-axis) vs. the poly(I:C)-activated condition (y-axis) for HPMECs (**C**) and HCAECs (**D**). Boxplots of eigengene expression and list of pathways overrepresented in the HPMEC p-green and p-sienna3 modules (**E**) and the HCAEC c-magenta and c-purple modules (**F**).

Given the above common observations from DEG-based pathway analysis in HPMECs and HCAECs, we performed further analysis to identify shared regulatory mechanisms between the two cell types that may underlie NMN-induced changes in gene expression during poly(I:C)-induced responses. To assess the regulatory transcriptome modules during poly(I:C) activation and investigate NMN dose effects, we performed weighted gene correlation network analysis (WGCNA). The HPMEC transcriptome data were clustered into 41 groups, and the HCAEC transcriptome data were clustered into 12 groups via WGCNA, as illustrated in the cluster dendrogram in Fig. 2A,B. The modules are named by a code indicating in which cell line the module was defined (p: HPMEC, c: HCAEC), followed by a color name, which is an arbitrarily assigned word to make it possible to refer to a module using an easily remembered code. The expression tendencies of each module were statistically assessed by correlation analysis with the traits defined in Supplementary Table 3: NMN dose levels (control, low, medium, and high) and poly(I:C) activation. Five modules out of 41 for HPMECs and 6 modules out of 12 for HCAECs (Supplementary Information 3) were identified as affected modules, with drastic changes in expression (*p* value < 1E−5) resulting from poly(I:C) induction. On the other hand, NMN dose trait analysis resulted in the identification of 2 modules for HPMECs and 1 module for HCAECs with significant expression changes. The comparison and overlap of gene members of modules between HPMECs and HCAECs are shown in a Sankey diagram (Supplementary Fig. 3) in which common genes are connected by lines between HPMECs (left) and HCAECs (right).

To assess the effect of high-dose NMN in non-poly(I:C)-activated samples and poly(I:C)-activated samples, we determined the fold change (FC) in expression levels of eigengenes for all modules between control and high-dose NMN, as presented in Fig. 2C,D; the x-axis represents the FC in non-poly(I:C)-activated samples, and the y-axis represents the FC in poly(I:C)-activated samples. The scatter plots enabled extraction of modules in which the effect of a high NMN dose is different between non-poly(I:C)-activated and poly(I:C)-activated conditions. In HPMECs, the eigengenes of the p-green and p-sienna3 modules had much larger fold-increases in the non-poly(I:C)-activated condition than in the poly(I:C)-activated condition (Fig. 2C). Similarly, the eigengenes of the c-magenta and c-purple modules are more upregulated (have a larger positive FC) in the non-poly(I:C)-activated condition than in the poly(I:C)-activated condition in HCAECs (Fig. 2D). In the HPMEC transcriptome data, the eigengene of the p-green module, *APLN*, was upregulated by NMN almost twice as much in the non-poly(I:C)-activated condition as in the poly(I:C)-activated condition; the eigengene of the HPMEC p-sienna3 module, *CTSS*, was upregulated by NMN more than twice as much in the non-poly(I:C)-activated condition as in the poly(I:C)-activated condition. We analyzed gene members of the HPMEC p-green and p-sienna3 modules with pathway enrichment and found that pathways related to mitochondrial oxidative phosphorylation were overrepresented in the p-green module and inflammatory pathways were overrepresented in the p-sienna3 module (Fig. 2E, Supplementary Information 3). Pathways similar to those in the HPMEC p-sienna3 module were found in the HCAEC c-magenta and c-purple modules, respectively (Fig. 2F, Supplementary Information 3).

**Figure 3:**
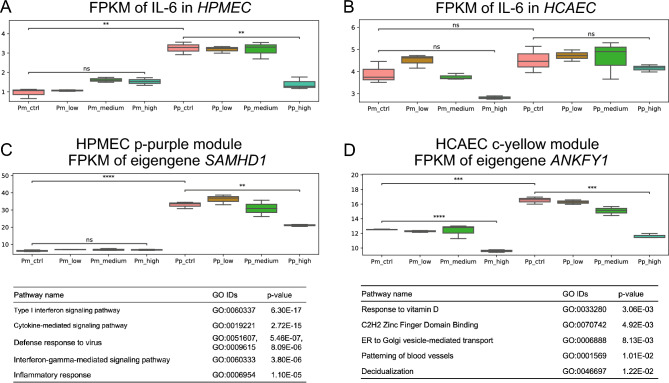
Functional enrichment analysis of different doses of NMN with or without poly(I:C) activation. Expression levels of *IL6* in HPMECs (**A**) and HCAECs (**B**). Expression levels of the eigengene (SAMHD1) for the HPMEC purple module (**C**) and the HCAEC yellow module (**D**).

Figure 3A,B show changes in the expression levels of *IL6* in HPMECs and HCAECs, respectively. In HPMECs, the expression level of *IL6* was significantly increased after poly(I:C) activation, and the level was decreased by high NMN in poly(I:C)-activated cell cultures (Fig. 3A). The expression level of *IL6* was slightly increased after NMN treatment in non-poly(I:C)-activated cell cultures. On the other hand, *IL6* expression was decreased by high-NMN treatment in both non-poly(I:C)-activated and poly(I:C)-activated conditions in HCAECs; these changes were, however, not statistically significant despite the clear expression increase by RT‒PCR. Interestingly, *IL6* was coexpressed with the members of the HPMEC p-purple module and HCAEC c-yellow module, which were extracted as modules in which NMN treatment reduced the expression in the poly(I:C)-activated condition more than in the poly(I:C)- condition. Figure 3C,D show changes in the expression levels in these modules of interest. The eigengenes of the HPMEC p-purple module and HCAEC c-yellow module were *SAMHD1* and *ANKFY1*, respectively, which had significant increases in expression upon poly(I:C) activation that were alleviated by high-dose NMN.

Next, the characteristics of gene members in the HPMEC p-purple module and HCAEC c-yellow module were assessed via pathway enrichment analysis (Fig. 3C,D, Supplementary Information 4). In HPMECs, the pathways overrepresented in the p-purple module were highly enriched in the inflammatory response, especially the type I interferon signaling pathway. Interestingly, the top 5 pathways overrepresented in the HCAEC c-yellow module are completely different from those overrepresented in the HPMEC p-purple module and appear to be unrelated to inflammation, such as vitamin D and blood vessel patterning pathways. Further investigation to identify similarities in the transcriptome expression profiles of HPMECs and HCAECs is necessary because we hypothesize that common pathways exist in response to poly(I:C) activation and NMN treatment in cells. Thus, assessment of the causal network among modules elucidated the potential regulatory mechanism underlying NMN-induced changes in the poly(I:C)-induced inflammatory response.

**Figure 4:**
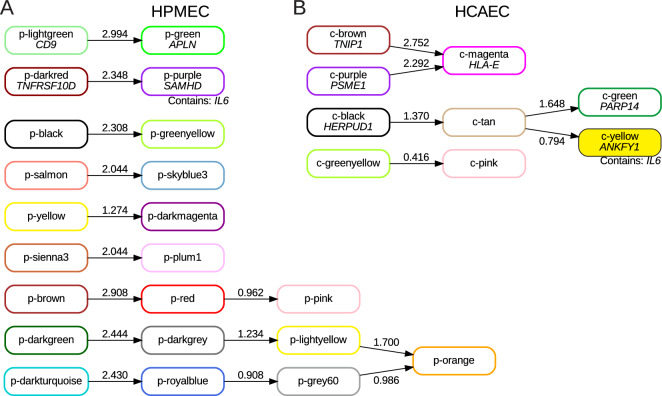
Predicted causal network of WGCNA eigengenes. Integers on the arrows represent the sum of “strength” computed via three different algorithms for Bayesian network inference. The HPMEC p-purple module is predicted to be regulated via the p-darkred module (**A**), and the HCAEC c-yellow module is predicted to be regulated through the c-black module via the tan module (**B**).

To this end, we applied Bayesian network analysis to infer potential regulatory mechanisms underlying the effect of NMN treatment on the poly(I:C)-induced inflammatory response. A general Bayesian network in which each node models the expression level of the eigengenes^13^ was utilized for causal network prediction. Figure 4A,B illustrate causal relationships among eigengenes of all WGCNA modules predicted by Bayesian network analysis; arrows represent causal regulation, and the values on the arrows are the sum of “strength” computed with three different algorithms in hybrid methods of *bnlearn* packages for HPMECs and HCAECs, respectively. For the predicted causal network, modules with the strongest “strength” include p-green, c-magenta, c-purple which were first presented in Fig. 2C–F and p-lightgreen, c-brown (Supplementary Fig. 4, Supplementary Information 5).

The eigengene of the HPMEC light p-green module, *CD9*, was upregulated by NMN treatment regardless of poly(I:C) activation; the eigengene of the HPMEC p-green module, *APLN*, had the same expression pattern as the HPMEC p-lightgreen module. We analyzed gene members of the HPMEC p-lightgreen and p-green modules for pathway enrichment and found that pathways related to mitochondrial oxidative phosphorylation were overrepresented in the p-green module and that antigen presentation-related pathways were overrepresented in the p-lightgreen module (Supplementary Fig. 4A). The HCAEC c-brown (Supplementary Fig. 4B) and c-purple (Fig. 2F) modules were predicted to regulate the c-magenta module (Fig. 4B); pathways related to antigen processing and presentation were overrepresented in HCAEC c-purple module gene members. Various pathways, including apoptosis pathways, alpha signaling pathways, and NF-kappa B signaling pathways, were overrepresented in the HCAEC c-magenta module. There were common pathways among HPMECs and HCAECs, such as antigen processing and mitochondrial oxidative phosphorylation, in the predicted networks. Apoptosis, alpha signaling pathways, and NF-kappa B signaling-related genes were enriched in the HCAEC c-magenta module, and these factors were specific to HCAECs and not included as pathways overrepresented in the HPMEC p-lightgreen and p-green modules.

**Figure 5:**
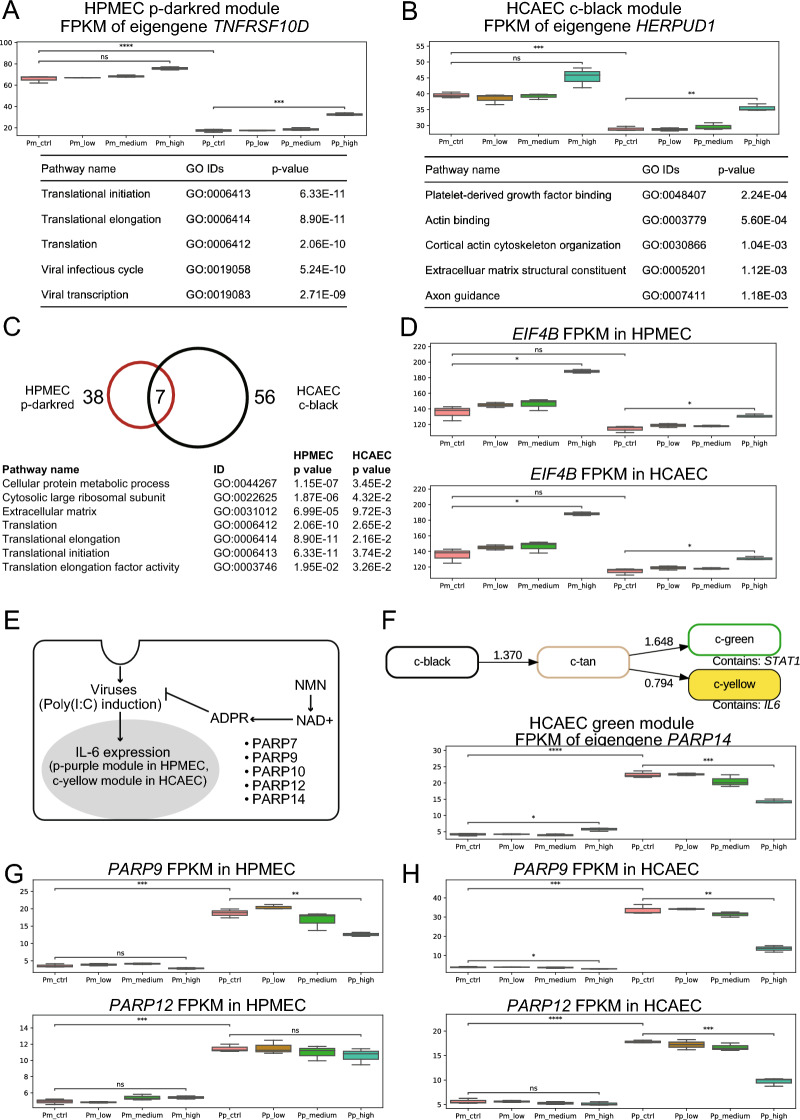
Inferred regulatory mechanism of NMN-mediated changes after poly(I:C) activation. (**A**) Expression level of the eigengene (TNFRSF10D) for the HPMEC p-darkred module and the results of functional enrichment analysis. (**B**) Expression level of the eigengene (HERPUD1) for the HCAEC c-black module and the results of functional enrichment analysis. (**C**) Common functional pathways between the HPMEC p-darkred module and HCAEC c-black module. (**D**) Boxplots for the expression levels of a representative common gene (EIF4B) in HPMECs and HCAECs. (**E**) A schematic model of the regulatory mechanism of the NMN-mediated decrease in *IL6* expression under poly(I:C)-activated conditions^25,28,29^. The poly(ADPribose) polymerase (PARP) family contains NAD(+)-consuming enzymes that transfer single ADPribose (ADPR) units from NAD(+) to polypeptide side chains to modify protein functions^21^. Because several PARPs are known to be induced by interferon, ADP-ribosylation is considered a host defense response, illustrated as a repression arrow from “ADPR” to “viruses”. Coronaviruses are known to encode an enzyme that removes ADP-ribosylation modifications^25^. (**F**) Predicted causal network among the c-black, c-tan, c-yellow, and c-green modules via Bayesian network analysis of eigengenes for HCAEC transcriptome data and an eigengene (PARP14) for the c-green module. (**G**) Boxplots of the expression levels of PARP9 and PARP12, both of which are included in the HPMEC p-purple module. (**H**) Boxplots of the expression levels of PARP9 and PARP12, both of which are included in the HCAEC c-green module.

Regarding the regulatory mechanisms underlying alleviation of the poly(I:C)-induced inflammatory response by NMN treatment, we note that the causal relationship of the HPMEC p-darkred module with the p-purple module in which *IL6* resides was relatively strong (Fig. 4A). The eigengene of the HPMEC p-darkred module was *TNFRSF10D*, a member of the TNF-receptor superfamily^20,21^; the expression level of *TNFRSF10D* was decreased by poly(I:C) activation and increased by NMN under poly(I:C)-activated conditions (Fig. 5A, Supplementary Information 6)^14,15^. In addition, translation and viral infection/transcription pathways were overrepresented in the HPMEC p-darkred module, indicating that application of NMN plays an important role in recovery of expression levels of inflammation-related genes, which were decreased by poly(I:C) activation. In HPMECs, Bayesian network analysis showed that NMN treatment triggers a direct causal relationship of translation and viral infection/transcription pathways with decreases in the expression levels of gene groups, including *IL6*.

On the other hand, the HCAEC c-yellow module was included in a predicted regulatory network consisting of four modules, the c-black, c-tan, c-yellow, and c-green modules (Fig. 4B). The eigengene of the HCAEC c-black module was *HERPUD1*, which plays an important role in the unfolded protein response (*UPR*) and the ER-associated protein degradation (ERAD) system^22,23^; the expression level of *HERPUD1* was increased by NMN under poly(I:C)-activated conditions (Fig. 5B). Pathways related to platelet-derived growth factor binding, actin binding, cortical actin cytoskeleton organization, extracellular matrix structural constituent, and axon guidance were overrepresented in the HCAEC c-black module. Notably, the platelet-derived growth factor binding pathway was overrepresented in HCAECs but not in HPMECs; this pathway may represent a response specifically observed in heart cells as a regulatory mechanism for alleviating inflammation via NMN treatment under poly(I:C)-activated conditions. This may indicate that NMN treatment may trigger an increase in the expression levels of genes related to the platelet-derived growth factor binding pathway under poly(I:C)-activated conditions to alleviate the inflammatory response in HCAECs.

Although the top five pathways overrepresented in putative regulatory gene groups were different between HPMECs and HCAECs (Fig. 5A,B, Supplementary Information 6), there were several pathways and genes that were common (Fig. 5C). The number of pathways enriched in the HPMEC p-darkred and HCAEC c-black modules and the number of common pathways in the two modules are shown in Fig. 5C. Among common pathways, three pathways are listed with p values in the HPMEC p-darkred module and HCAEC c-black module in the panels of Fig. 5C; these pathways include “cellular protein metabolic process”, “cytosolic large ribosomal subunit” and “extracellular matrix”. *EIF4B* is one of the common genes between the HPMEC p-darkred module and the HCAEC c-black module and involved in the common pathways listed in Fig. 5C; other common genes were *RPS3A*, *RPL4*, *RPL7A*, and *RPL17*. NMN treatment generally increased the expression level of *EIF4B* in both HPMECs and HCAECs, except for the poly(I:C)-activation condition in HCAECs, for which the expression level was slightly decreased but not statistically significant (Fig. 5D).

*EIF4B* stands for eukaryotic translation initiation factor 4B, and EIF4B is known as a cofactor of RNA helicase EIF4A that unwinds local secondary structures and creates a ribosome landing pad on mRNA^18^. Although the relationship among the poly(I:C)-induced inflammatory response, NMN treatment, and the expression level of *EIF4B* has not yet been determined, it is worth noting that EIF4B plays an important role in translation initiation together with EIF4A, and *EIF4B* is regulated by two major proto-oncogenic signaling pathways, Ras-MAPK and PI3-K/mTOR, which are important targets for cancer treatment^24^.

The PARP family includes major NAD(+)-consuming enzymes that mediate the host defense response via ADP-ribosylation. Reportedly, coronaviruses encode an enzyme that removes ADP-ribosylation modifications, which may lead to hyperinflammation^25^. Poly(I:C) activation increased the expression levels of genes included in the same module as *IL6*. NMN treatment decreased the expression levels of genes in the poly(ADP-ribose) polymerase (PARP) family. From these results, we hypothesized a mechanism for NMN-driven alleviation of the poly(I:C)-induced inflammatory response (Fig. 5E). NMN treatment increases the intracellular NAD(+) concentration, and the PARP family consumes NAD(+) to mediate the host defense response to viruses (poly(I:C) activation) via ADP-ribosylation. Consequently, ADP-ribosylation alleviates the inflammatory response triggered by poly(I:C) activation.

In HCAECs, *IL6* was included in the c-yellow module, and *STAT1* was included in the c-green module (Fig. 5F). The eigengene of the HCAEC c-green module was *PARP14,* and the expression level of *PARP14* was decreased by NMN treatment in a concentration-dependent manner under poly(I:C)-activated conditions. In both HPMECs and HCAECs, the expression levels of *PARP9* and *PARP12* were decreased by NMN treatment under poly(I:C)-activated conditions only (Fig. 5G,H ). These results further support our hypothesis that PARP-dependent ADP-ribosylation may repress the inflammatory response caused by poly(I:C) activation^26^ (Fig. 5E). Poly(I:C) activation significantly increased the expression level, and NMN treatment alleviated this increase in the expression level, indicating that NMN treatment has a significant effect on the poly(I:C)-induced inflammatory response.

Sirtuins are another group of NAD+-consuming enzymes that are known to have antiviral activity^27^. Unlike the PARP family, there was no significant change of any of the seven sirtuins in the two tested cell lines upon poly(I:C) activation in the controls (Supplementary Fig. 5, 6). Nevertheless, NMN at a high dose significantly downregulated *SIRT6* in both HCAECs and HPMECs and *SIRT1* in HCAECs under poly(I:C)-activated conditions (Supplementary Fig. 6). *SIRT5* is significantly upregulated in HCAECs under poly(I:C)-activated as well as non-poly(I:C)-activated conditions, and significantly upregulated in HPMECs under poly(I:C)-activated conditions (Supplementary Fig. 5).

## Discussion

A major finding of this study is that expression profiling revealed the anti-inflammatory activity of NMN and hinted at the underlying pathways. Poly(I:C) mimics viral infection and induces an inflammatory response^8^. *IL6* is an important marker of inflammation^30^ and is upregulated in numerous diseases, including cardiovascular^31^ and cardio-cerebrovascular^32^ diseases. We observed an increase in *IL6* expression by RT‒PCR and RNA-seq after poly(I:C) intervention and a significant reduction in *IL6* expression after subsequent NMN treatment in the HPMECs. Dysregulation of *IL6* expression was not significant in HCAECs. Because gene expression changes are time-dependent and RNA-seq can be vulnerable to noise, we applied WGCNA, as gene clusters are more robust than differential expression profiling of single genes^14^. In HCAECs, even this clustering approach did not immediately support the existence of NMN-mediated alleviation of poly(I:C)-induced inflammation because the gene cluster containing *IL6* was enriched in GO pathways without obvious relevance to inflammation. Therefore, we next performed Bayesian network analysis, which revealed regulatory control by the HCAEC c-black module via the c-tan module and the c-yellow module, which contains *IL6*. Intriguingly, the platelet-derived growth factor (PDGF) binding pathway was the top overrepresented pathway in the c-black module. PDGFs are tightly connected to inflammation^33^. The most representative member of the HCAEC c-black module, its eigengene *HERPUD1*, was significantly upregulated by poly(I:C). Thus, a systems biology approach provided support for the existence of a causal link between NMN treatment and reduced inflammation via PDGFs in both HPMECs and HCAECs.

The anti-inflammatory activity of NMN suggests that it may be a therapeutic intervention for diseases with an inflammatory component. This includes both cardiovascular diseases, many of which are now understood to be closely linked to inflammation^34^, and viral infections, notably SARS-CoV-2, which can trigger the cytokine storm^35^. The cytokine storm is an overreaction of the immune system characterized by a flood of interleukins, with severe consequences^36^. The results of this study support the idea that NMM could be used as a nutraceutical or as a drug in these disease contexts to reduce atherosclerosis or resolve the cytokine storm. A recent study of SARS-CoV-2-infected mice suggests that NMN might alleviate the immune storm triggered by the virus by targeting the genes *Cd200r3* and *Cd200r4*^6^. We note that the correlation of doses explored in this study and the clinically effective or tolerable levels of NMN for dietary or therapeutic intervention has not been discussed.

The PARP and sirtuin families comprise pleiotropic proteins with diverse roles, including roles in the DNA damage response^37^, and play functional roles in response to viral infection and inflammation^7,25,26^. Specific PARP and sirtuin family members were revealed to be dysregulated by poly(I:C) intervention and NMN treatment in this research, as discussed in the following two paragraphs.

The differential expression of PARP9, 12, and 14 is intriguing, as these proteins play a central role in the “NAD+ battlefield” concept^7^. These three PARPs consume NAD+ to enact posttranslational modifications^25^, while certain viruses encode functions to undo these modifications^29^. PARP9, 12, and 14 were significantly upregulated by poly(I:C) in both cell lines. NMN treatment significantly downregulated PARP9 and 12 in HCAECs and PARP9 in HPMECs (Fig. 5G,H ). A possible interpretation could be that as NAD+ becomes more abundant because of NMN supplementation, the pool of PARPs is more effective in inducing posttranslational modifications. Exploiting an abundance of NAD+ might cost the cell less energy overall than trying to counteract the viral activity by upregulating the PARPs, as it does in the absence of NAD+-boosting NMN.

SIRT5 was one of the sirtuins we found to be differentially upregulated after NMN treatment in both poly(I:C)-activated and non-poly(I:C)-activated conditions in HCAECs, and upregulated after NMN treatment in poly(I:C)-activated conditions in HPMECs. SIRT5 is one of the sirtuins localized to mitochondria^38^, and we found that the expression of multiple WGCNA modules related to mitochondrial metabolism increased after NMN, as expected^39^, confirming the connection between NMN and mitochondrial metabolism. Conflicting reports attribute proviral^40^ and antiviral^41^ activity to SIRT5, illustrating how challenging and context-dependent it is to interpret changes in direction (increase vs. decrease in expression) and functional consequences.

While transcriptome profiling is a powerful tool to study the mechanisms and outcomes of NMN intervention in the context of poly(I:C)-induced inflammation, experimental measures of protein abundance, particularly IL6, in response to poly(I:C), the protein abundance of differentially expressed PARPs and sirtuins, and the assumed NAD+ increase^42^ (or NAMPT enzymatic activity) after NMN treatment would increase support for the findings of this study. Other studies have indeed shown that IL6 secretion increases after poly(I:C) treatment in HPMECs^43^ and in a panel of endothelial cell lines, including HCAECs^44^, and that NMN restores cellular levels of NAD+ , which are decreased in LPS-activated macrophages^13^; these findings are promising for future investigations of the working hypothesis proposed in this study.

## Supplementary Information

Supplementary Information 1.

Supplementary Information 2.

Supplementary Information 3.

Supplementary Information 4.

Supplementary Information 5.

Supplementary Information 6.

Supplementary Table 1.

Supplementary Table 2.

Supplementary Table 3.

Supplementary Information 10.

## Data Availability

The raw and processed RNA-seq data generated and analyzed in this study are available at NCBI GEO under accession number GSE233679 at https://www.ncbi.nlm.nih.gov/geo/query/acc.cgi?acc=GSE233679.

## Code availability

All codes used in this study including WGCNA, pathway enrichment analysis and Bayesian network analysis are available at the GitHub repository (https://github.com/Yachie-SBI/SciRep202307).

## References

[1] Mouchiroud L (2013). The NAD(+)/sirtuin pathway modulates longevity through activation of mitochondrial UPR and FOXO signaling. Cell.

[2] Mills KF (2016). Long-term administration of nicotinamide mononucleotide mitigates age-associated physiological decline in mice. Cell Metab..

[3] Reiten OK, Wilvang MA, Mitchell SJ, Hu Z, Fang EF (2021). Preclinical and clinical evidence of NAD+ precursors in health, disease, and ageing. Mech. Ageing Dev..

[4] Fang EF (2019). Mitophagy inhibits amyloid-β and tau pathology and reverses cognitive deficits in models of Alzheimer’s disease. Nat. Neurosci..

[5] Fang EF (2019). NAD+ augmentation restores mitophagy and limits accelerated aging in Werner syndrome. Nat. Commun..

[6] Jiang Y (2022). Treatment of SARS-CoV-2-induced pneumonia with NAD+ and NMN in two mouse models. Cell Discov..

[7] Brenner C (2022). Viral infection as an NAD+ battlefield. Nat. Metab..

[8] Fortier M-E (2004). The viral mimic, polyinosinic:polycytidylic acid, induces fever in rats via an interleukin-1-dependent mechanism. Am. J. Physiol. Regul. Integr. Comp. Physiol..

[9] Matsumoto M, Kikkawa S, Kohase M, Miyake K, Seya T (2002). Establishment of a monoclonal antibody against human Toll-like receptor 3 that blocks double-stranded RNA-mediated signaling. Biochem. Biophys. Res. Commun..

[10] Alexopoulou L, Holt AC, Medzhitov R, Flavell RA (2001). Recognition of double-stranded RNA and activation of NF-kappaB by Toll-like receptor 3. Nature.

[11] Yamamoto M (2003). Role of adaptor TRIF in the MyD88-independent toll-like receptor signaling pathway. Science.

[12] Arunachalam PS (2020). Systems biological assessment of immunity to mild versus severe COVID-19 infection in humans. Science.

[13] Liu J (2021). Nicotinamide mononucleotide alleviates LPS-induced inflammation and oxidative stress via decreasing COX-2 expression in macrophages. Front. Mol. Biosci..

[14] Langfelder P, Horvath S (2008). WGCNA: an R package for weighted correlation network analysis. BMC Bioinform..

[15] Martin JA, Wang Z (2011). Next-generation transcriptome assembly. Nat. Rev. Genet..

[16] Pedregosa, F. *et al*. Scikit-learn: Machine learning in Python. (2012) 10.48550/ARXIV.1201.0490.

[17] Strimmer K (2008). fdrtool: A versatile R package for estimating local and tail area-based false discovery rates. Bioinform. Oxf. Engl..

[18] Benjamini Y, Hochberg Y (1995). Controlling the false discovery rate: A practical and powerful approach to multiple testing. J. R. Stat. Soc. Ser. B Methodol..

[19] Agrahari R (2018). Applications of Bayesian network models in predicting types of hematological malignancies. Sci. Rep..

[20] Marsters SA (1997). A novel receptor for Apo2L/TRAIL contains a truncated death domain. Curr. Biol. CB.

[21] Pan G, Ni J, Yu G, Wei YF, Dixit VM (1998). TRUNDD, a new member of the TRAIL receptor family that antagonizes TRAIL signalling. FEBS Lett..

[22] van Laar T (2000). The novel MMS-inducible gene Mif1/KIAA0025 is a target of the unfolded protein response pathway. FEBS Lett..

[23] Kokame K, Agarwala KL, Kato H, Miyata T (2000). Herp, a new ubiquitin-like membrane protein induced by endoplasmic reticulum stress. J. Biol. Chem..

[24] Shahbazian D, Parsyan A, Petroulakis E, Hershey J, Sonenberg N (2010). eIF4B controls survival and proliferation and is regulated by proto-oncogenic signaling pathways. Cell Cycle Georget. Tex..

[25] Alhammad YMO, Fehr AR (2020). The viral macrodomain counters host antiviral ADP-ribosylation. Viruses.

[26] Fehr AR (2020). The impact of PARPs and ADP-ribosylation on inflammation and host-pathogen interactions. Genes Dev..

[27] Koyuncu E (2014). Sirtuins are evolutionarily conserved viral restriction factors. mBio.

[28] Heer CD (2020). Coronavirus infection and PARP expression dysregulate the NAD metabolome: An actionable component of innate immunity. J. Biol. Chem..

[29] Cohen MS (2020). Interplay between compartmentalized NAD+ synthesis and consumption: A focus on the PARP family. Genes Dev..

[30] Tanaka T, Narazaki M, Kishimoto T (2014). IL-6 in inflammation, immunity, and disease. Cold Spring Harb. Perspect. Biol..

[31] Feng Y (2022). The role of interleukin-6 family members in cardiovascular diseases. Front. Cardiovasc. Med..

[32] Su J-H (2021). Interleukin-6: A novel target for cardio-cerebrovascular diseases. Front. Pharmacol..

[33] von Hundelshausen P, Weber C (2007). Platelets as immune cells: Bridging inflammation and cardiovascular disease. Circ. Res..

[34] Libby P (2006). Inflammation and cardiovascular disease mechanisms. Am. J. Clin. Nutr..

[35] Hu B, Huang S, Yin L (2021). The cytokine storm and COVID-19. J. Med. Virol..

[36] Fajgenbaum DC, June CH (2020). Cytokine storm. N. Engl. J. Med..

[37] Sousa FG (2012). PARPs and the DNA damage response. Carcinogenesis.

[38] Kumar S, Lombard DB (2018). Functions of the sirtuin deacylase SIRT5 in normal physiology and pathobiology. Crit. Rev. Biochem. Mol. Biol..

[39] Stein LR, Imai S (2012). The dynamic regulation of NAD metabolism in mitochondria. Trends Endocrinol. Metab. TEM.

[40] Zheng M, Schultz MB, Sinclair DA (2022). NAD+ in COVID-19 and viral infections. Trends Immunol..

[41] Shang J, Smith MR, Anmangandla A, Lin H (2021). NAD+-consuming enzymes in immune defense against viral infection. Biochem. J..

[42] Braidy N, Villalva MD, Grant R (2021). NADomics: Measuring NAD+ and related metabolites using liquid chromatography mass spectrometry. Life Basel Switz..

[43] Ocaña-Macchi M (2009). Hemagglutinin-dependent tropism of H5N1 avian influenza virus for human endothelial cells. J. Virol..

[44] Emi-Sugie M (2020). Robust production of IL-33 and TSLP by lung endothelial cells in response to low-dose dsRNA stimulation. J. Allergy Clin. Immunol..

