# Supplementary material for: Nicotinamide mononucleotide (NMN) alleviates the poly(I:C)-induced inflammatory response in human primary cell cultures
Source: Sci Rep. 2023 Jul 20;13:11765. doi: 10.1038/s41598-023-38762-x (PMC10359400; doi:10.1038/s41598-023-38762-x)
Supplement: Supplementary file 10 — Supplementary Information 10. [file 41598_2023_38762_MOESM10_ESM.pdf]

# Supplementary Material

## Nicotinamide mononucleotide (NMN) alleviates the poly(I:C)-induced inflammatory response in human primary cell cultures

Hitomi Sano, Anton Kratz, Taiko Nishino, Haruna Imamura, Yuki Yoshida, Noriaki Shimizu, Hiroaki Kitano and Ayako Yachie

## 1 List of Supplementary Figures

**Supplementary Figure 1.** Images of representative cells from each condition at the time of RNA isolation.

**Supplementary Figure 2.** PCA plot for FPKM transcriptome data with poly(I:C) (2.5 μg/mL) induction (red) and without poly(I:C) induction (blue) in HPMEC (A) and HCAEC (B).

**Supplementary Figure 3.** Sankey diagram illustrating common genes between HPMEC and HCAEC.

**Supplementary Figure 4.** Boxplots for additional eigengenes and list of pathways overrepresented in selected modules in HPMEC (A) and HCAEC (B).

**Supplementary Figure 5.** Gene expression of the seven sirtuin genes in HPMEC cells under different poly(I:C) conditions and NMN doses.

**Supplementary Figure 6.** Gene expression of the seven sirtuin genes in HCAEC cells under different poly(I:C) conditions and NMN doses.

## 2 List of Supplementary Tables

**Supplementary Table 1.** List of primers.

**Supplementary Table 2.** PCA loadings.

**Supplementary Table 3.** Traits for WGCNA.

## 3 List of Supplementary Information

**Supplementary Information 1.**

List of top 20 genes up-regulated by high-NMN in poly(I:C)-induced HPMEC.

List of top 20 genes down-regulated by high-NMN in poly(I:C)-induced HPMEC.

List of top 20 genes down-regulated by high-NMN in poly(I:C)-induced HCAEC.

List of top 20 genes up-regulated by high-NMN in poly(I:C)-induced HCAEC.

**Supplementary Information 2.**

Gene Ontology pathways enriched in up- and downregulated genes of HPMEC and HCAEC.

**Supplementary Information 3.**

Module-trait relationships (WGCNA p-values) in HPMEC.

Module-trait relationships (WGCNA p-values) in HCAEC.

Gene Ontology pathways overrepresented in the p-sienna3, p-green, c-magenta, c-purple modules. These are the full lists of which only excerpts are shown in Figure 2 E, F.

**Supplementary Information 4.**

Gene Ontology pathways overrepresented in the p-purple, c-yellow modules. These are the full lists of which only excerpts are shown in Figure 3 C, D.

**Supplementary Information 5.**

Gene Ontology pathways overrepresented in the p-lightgreen, c-brown modules. These are the full lists of which only excerpts are shown in Supplementary Figure 4.

**Supplementary Information 6.**

Gene Ontology pathways overrepresented in the p-darkred, c-black modules. These are the full lists of which only excerpts are shown in Figure 5 A, B.

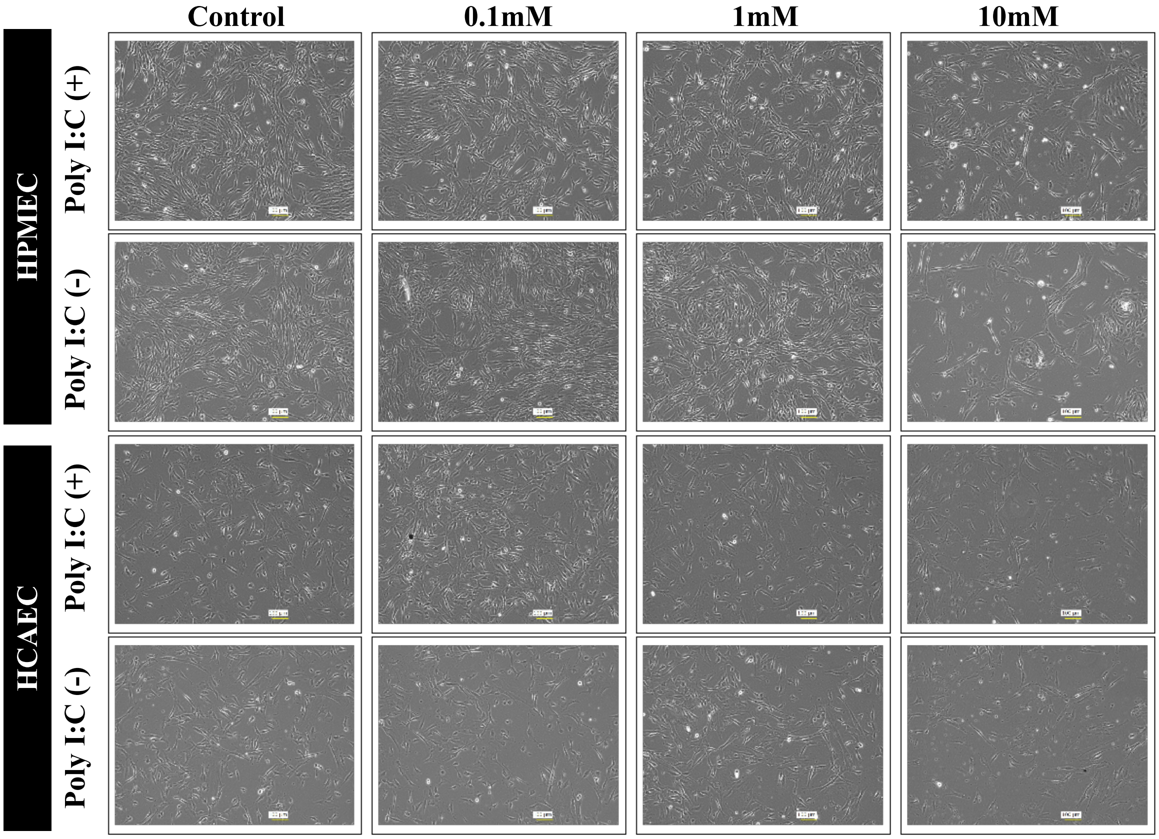


**Supplementary Figure 1:** Images of representative cells from each condition at the time of RNA isolation. Yellow bar 100 µm.

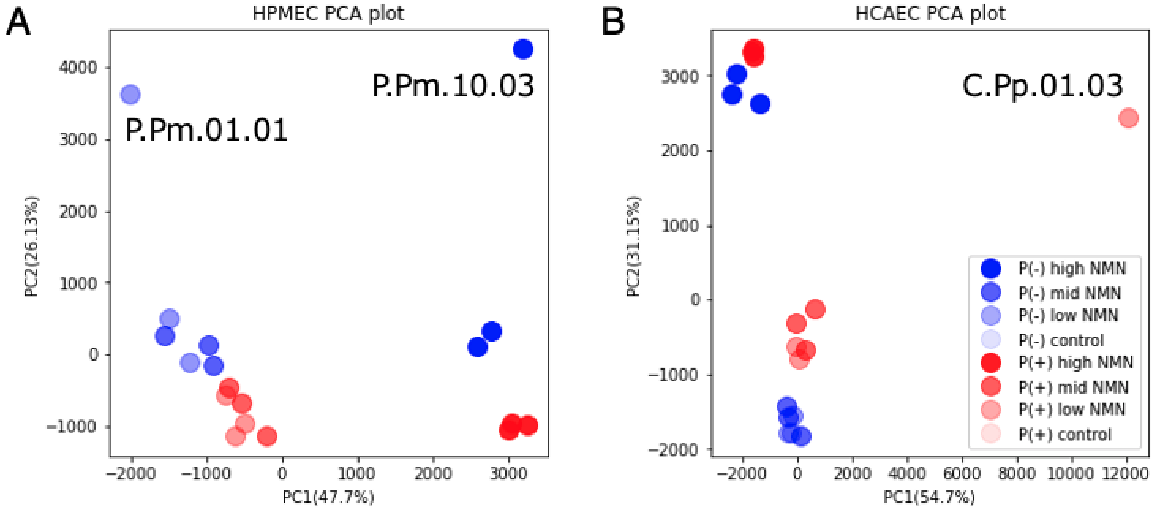


**Supplementary Figure 2:** PCA plot for FPKM transcriptome data with poly(I:C) (2.5μg/mL) induction (red) and without poly(I:C) induction (blue) in HPMEC (A) and HCAEC (B). Pp: Poly(I:C)+. Pm: Poly(I:C)-. low: 0.1 mM, mid: 1.0 mM, high: 10 mM NMN.

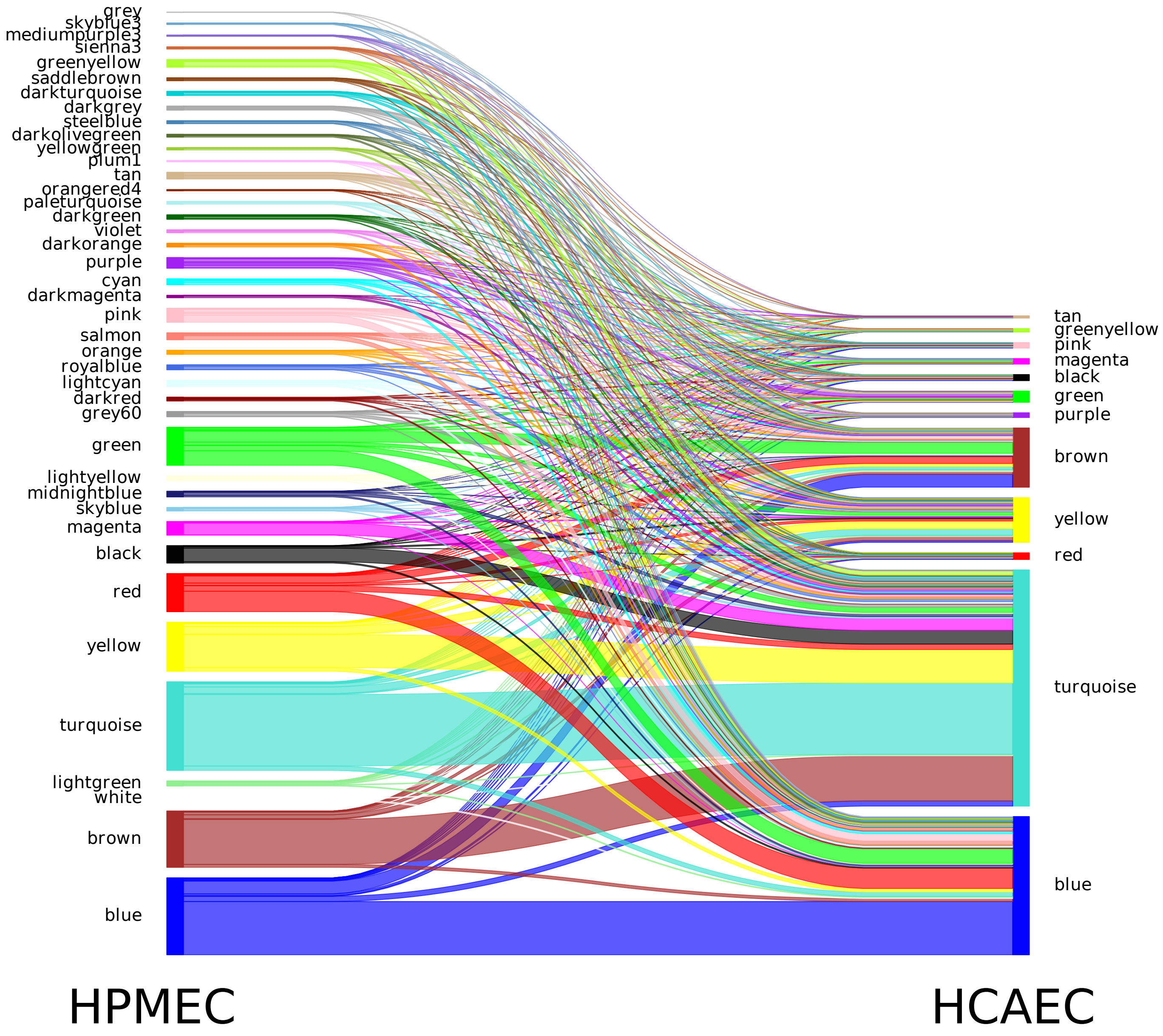


**Supplementary Figure 3:** Sankey diagram illustrating common genes between HPMEC and HCAEC.

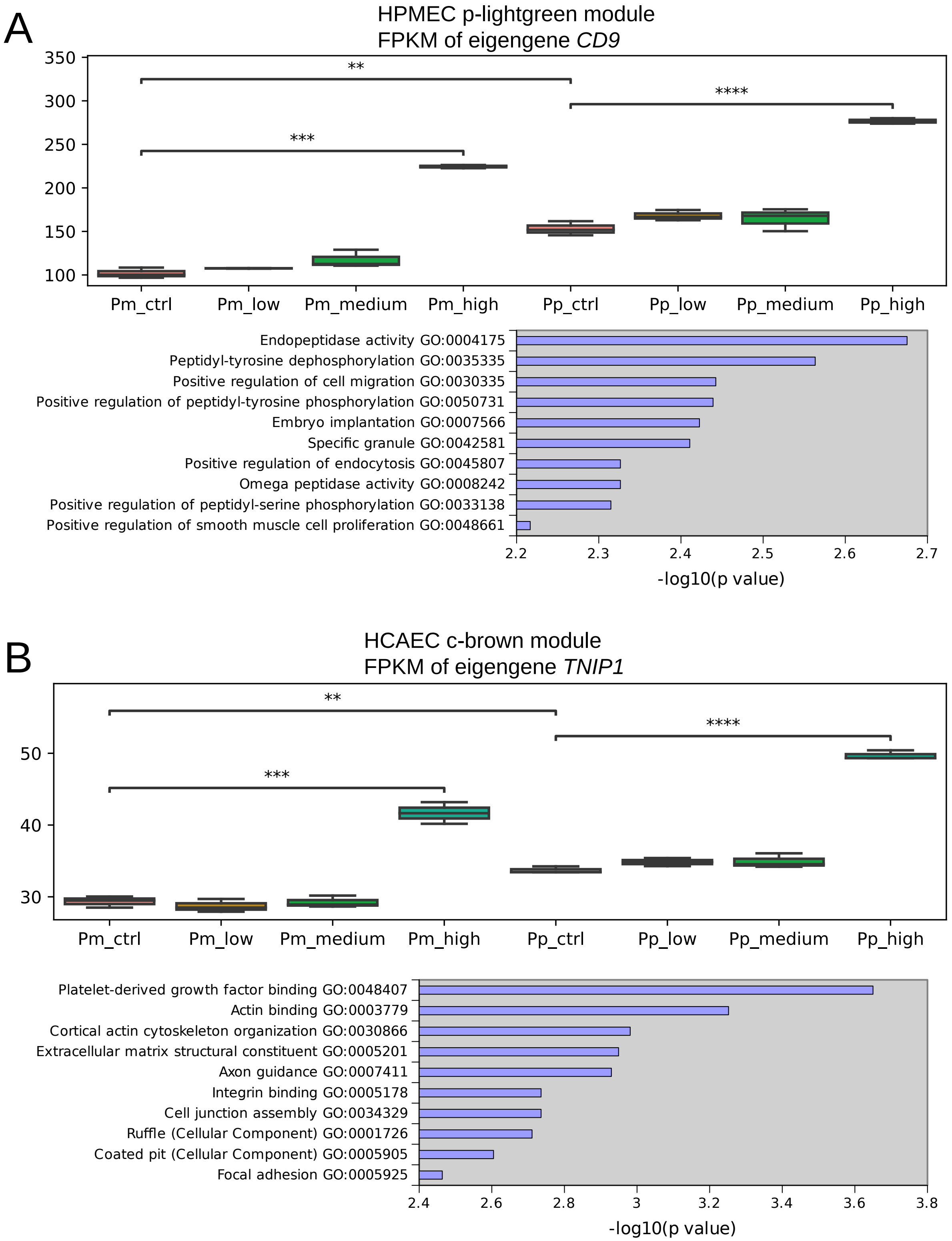


**Supplementary Figure 4:** Boxplots for eigengenes and list of pathways overrepresented in selected modules in HPMEC (A) and HCAEC (B). Pp: Poly(I:C)+. Pm: Poly(I:C)-. low: 0.1 mM, medium: 1.0 mM, high: 10 mM NMN.

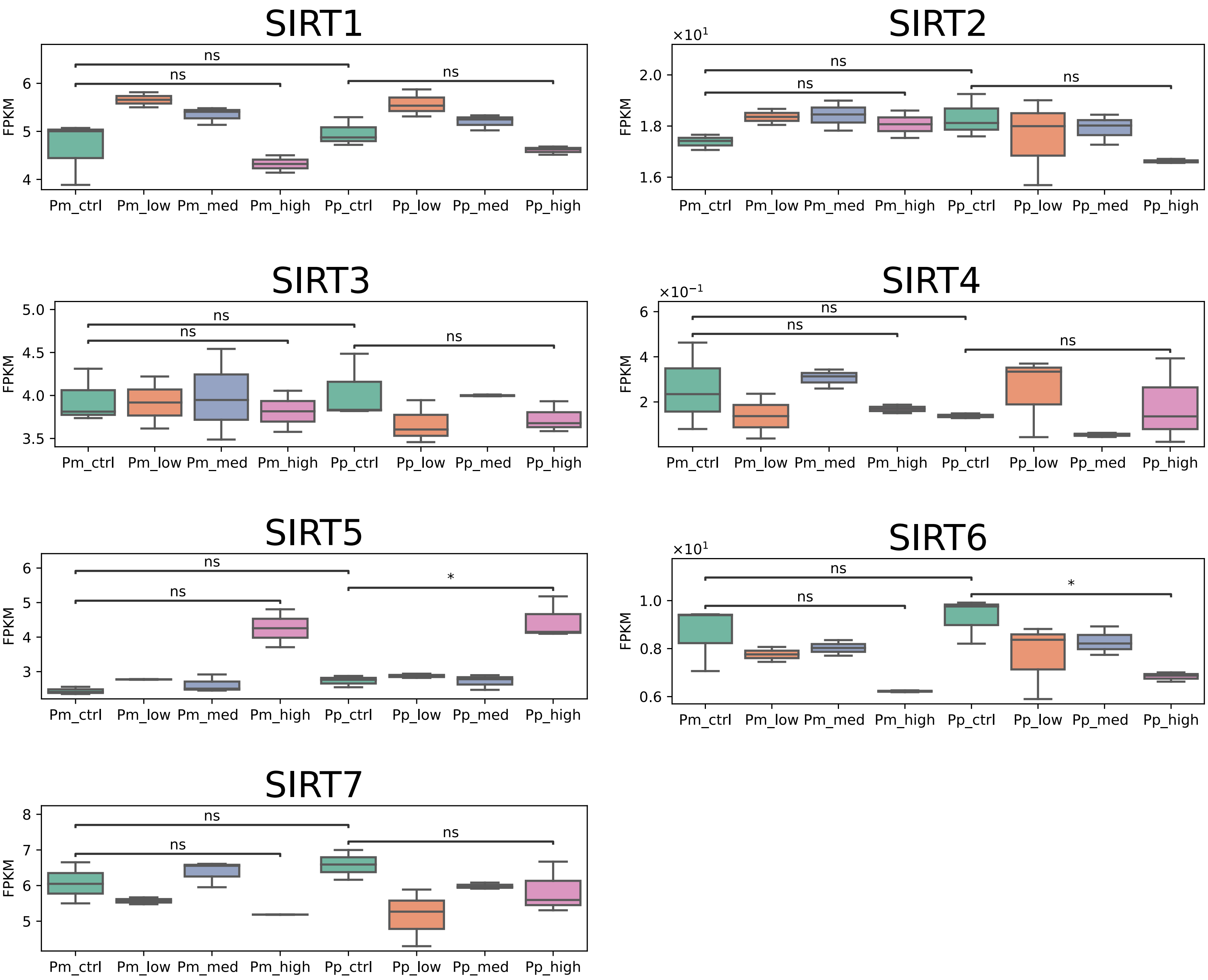


**Supplementary Figure 5:** Gene expression of the seven sirtuin genes in HPMEC cells under different poly(I:C) conditions and NMN doses. Pp: Poly(I:C)+. Pm: Poly(I:C)-. low: 0.1 mM, med: 1.0 mM, high: 10 mM NMN.

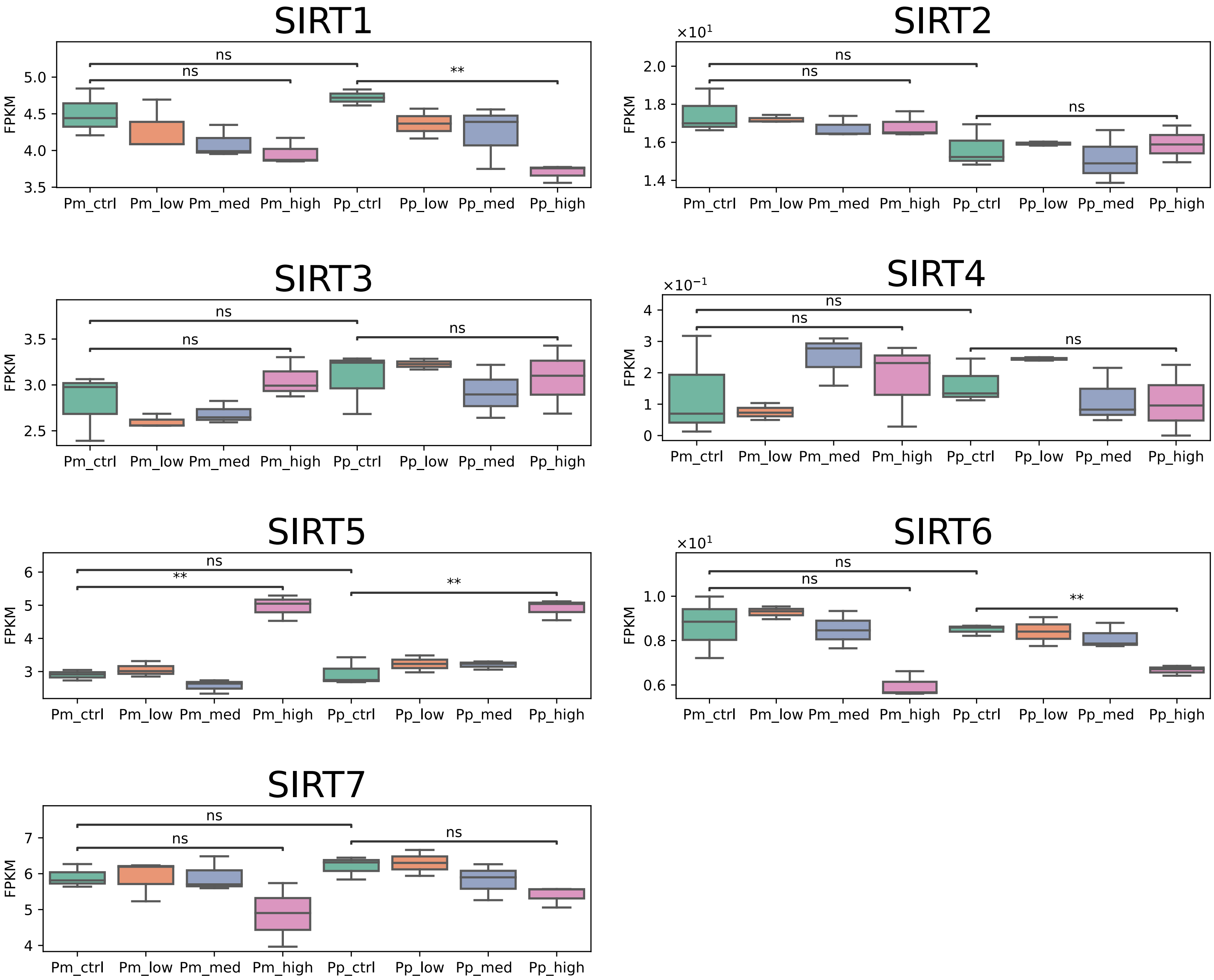


**Supplementary Figure 6:** Gene expression of the seven sirtuin genes in HCAEC cells under different poly(I:C) conditions and NMN doses. Pp: Poly(I:C)+. Pm: Poly(I:C)-. low: 0.1 mM, med: 1.0 mM, high: 10 mM NMN.
